# Mifepristone: Reflections on its Early Clinical Development for Medical Abortion

**DOI:** 10.5041/RMMJ.10576

**Published:** 2026-04-26

**Authors:** Irving M. Spitz

**Affiliations:** 1 Adjunct Professor of Medicine, Joan and Sanford I. Weill Medical College of Cornell University, New York City, NY, USA; 2 Emeritus Professor of Endocrinology, Ben Gurion University of the Negev, Beer-Sheva, Israel; 3 Institute of Hormone Research, Jerusalem, Israel

**Keywords:** Antiprogestins, clinical development, medical abortion, mifepristone, RU-486

## Abstract

Mifepristone (RU-486), the first clinically effective progesterone antagonist, was initially identified as having antiglucocorticoid activity. Although early interest focused on its role as a glucocorticoid receptor antagonist, recognition of its antiprogesterone effects led to studies of its role in pregnancy termination. This article reviews the steps leading to its use as an abortifacient, from its initial identification for this indication and draws on the author’s direct involvement in the field with reflections on his contribution to the early clinical development of mifepristone. These studies established the efficacy of mifepristone in pregnancy termination. Unfortunately, its wider therapeutic applications have received limited attention.

## INTRODUCTION

Mifepristone (RU-486), the first clinically effective progesterone antagonist, represents a landmark advance in reproductive endocrinology. Initially developed as a glucocorticoid antagonist, its antiprogesterone activity was identified in 1982, prompting investigation of its role in pregnancy termination. This article reviews the early clinical development of mifepristone in the United States (US), including studies establishing its safety, metabolism, and lack of clinically significant adrenal suppression at doses used for medical abortion. Early trials showed limited efficacy with mifepristone alone; however, combination therapy with prostaglandins—ultimately misoprostol—markedly improved outcomes. Results from a pivotal multicenter US clinical trial formed the basis for Food and Drug Administration (FDA) approval in 2000. Subsequent protocol refinements expanded access and simplified administration. This review outlines the role played by the author in the early clinical studies in establishing its safety, pharmacologic profile and clinical utility. Despite its success as an abortifacient, mifepristone’s broader therapeutic potential remains incompletely explored.

## EARLY STUDIES AND CHALLENGES

In the 1970s, the French pharmacological company Roussel Uclaf launched a research project to identify glucocorticoid antagonists. In 1980, Georges Teutsch, the senior chemist in the company, synthesized RU-38486, which proved to be a potent glucocorticoid antagonist.^1^ At the Sixth International Congress on Hormonal Steroids held in Jerusalem in September 1982, Daniel Philibert reported that RU-38486 was also a potent progesterone antagonist.^2^ This compound, later abbreviated to RU-486, is now currently known by its generic name, mifepristone.

At that time, I was recruited by Wayne Bardin, director of the Center for Biomedical Research at the Population Council in New York, to join the Council as a senior scientist and director of clinical research. At the Jerusalem conference, Bardin and I met with several scientists from Roussel Uclaf, including Daniel Philibert. Fascinated by the potential clinical applications of these compounds, I arranged to receive a supply of mifepristone from Philibert to begin clinical studies in the US.

After joining the Population Council in October 1982, I directed the initial studies and development of mifepristone in the US, collaborating with a highly skilled team of scientists at the Population Council and other prominent research institutions both in the US and internationally.

Since mifepristone acts as both an antiglucocorticoid and an antiprogestin, we needed to ensure that adrenal function would remain uncompromised when the drug was administered. Through a series of studies conducted in both humans and animals, we confirmed that the doses of mifepristone required for pregnancy interruption did not adversely affect adrenal function. In fact, achieving a desired antiglucocorticoid effect required an order of magnitude higher dose than those used for pregnancy termination.^3^ Additionally, our studies demonstrated that both single and multiple doses of mifepristone were well tolerated, with no significant adverse effects. Furthermore, we investigated the metabolism of mifepristone, its physiological properties, and the effects of long-term administration.^3^

Our initial studies on pregnancy termination focused on women with amenorrhea of less than 49 days. We tested various dose schedules of mifepristone alone, but success rates ranged from only 50% to 86%, indicating that mifepristone alone was not a sufficiently effective abortifacient.^3^

It was well known that prostaglandins and their analogs could induce cervical softening, stimulate uterine contractions, and, in some cases, terminate pregnancy.^4^ Combining mifepristone with a prostaglandin administered 48 hours later significantly enhanced its efficacy as an abortifacient.^3^ Initially, prostaglandin analogs such as sulprostone (a prostaglandin E2 analog) and gemeprost (a prostaglandin E1 analog) were used. Subsequently it transpired that misoprostol (Cytotec; GD Searle & Company, Skokie, IL, USA), an FDA-approved E1 prostaglandin analog used to prevent gastric ulcers in patients taking anti-inflammatory drugs, was the most effective and safe prostaglandin analog for use in pregnancy termination. Misoprostol is now the standard choice for this purpose.^3^

At the time, conducting clinical studies in pregnancy termination in the US posed substantial practical challenges. Abortion providers faced threats of violence, including arson and bombings of clinics, and targeted attacks on physicians and clinic staff.^5^ There were even murders or attempted murders of physicians who provided abortions.^6^

## CLINICAL TRIALS AND FDA APPROVAL

In the early 1990s, efforts to register mifepristone for medical pregnancy termination gained renewed momentum. With that support, we initiated the first large, multicenter US clinical trial evaluating mifepristone in combination with misoprostol for pregnancy termination.

In this trial, we administered 600 mg of mifepristone followed by 400 μg of misoprostol 48 hours later to 2,121 women seeking pregnancy termination at 17 centers across the US.^7^ The outcomes were as follows: termination was achieved in 92% of women with amenorrhea under 49 days, 83% of those with amenorrhea between 49 and 56 days, and 77% of those with amenorrhea between 57 and 63 days. Among those with successful termination, 49% experienced pregnancy termination within 4 hours of taking misoprostol, and, by 24 hours, 75% had expelled the conceptus. Failures increased with the duration of pregnancy, with ongoing pregnancies rising from 1% in the <49-days group to 9% in the 57–63-days group. Adverse effects, including abdominal pain, nausea, vomiting, diarrhea, and vaginal bleeding, also became more common with advancing gestational age. Hospitalization, surgical intervention, or intravenous fluids were required for 2% of participants in the <49-days group and 4% in the two latter groups.^7^

This extensive US clinical trial demonstrated the efficacy and safety of mifepristone in combination with misoprostol for pregnancy termination, particularly in women with pregnancies of less than 49 days. The data formed the core of the New Drug Application (NDA) submitted to the FDA, a key step in seeking approval for marketing and distribution of a new drug in the US. In September 2000, after a lengthy and rigorous process, the FDA approved mifepristone for the medical termination of pregnancy at up to seven weeks of gestation.

In our large US study, participants were required to attend three clinic visits. On Day 1, women underwent clinical assessment by a licensed physician and ingested mifepristone. On Day 3, they returned to ingest misoprostol under observation and were monitored for four hours. On Day 15, the treatment outcome was assessed.^7^

Early clinical experience with medical abortion revealed that, although rare, serious complications could occur, including bleeding, unrecognized ectopic pregnancies at the time of treatment, and, in some very infrequent cases, severe infections and death.^8^ These complications were often associated with variations in misoprostol dosing and routes of administration. Hence, these potential risks justified the stringent requirements in our study.^7^

Over time, as familiarity with this regimen increased, the FDA simplified these protocols. Today, mifepristone and misoprostol can be safely administered at up to 70 days (10 weeks) of pregnancy. The FDA recommends 200 mg of mifepristone followed 24 to 48 hours later by 800 μg of misoprostol, administered buccally.^9^ Alternative routes, such as sublingual or vaginal administration, are also options for misoprostol.

In 2021, the FDA lifted the in-person dispensing requirement for mifepristone, allowing distribution through certified pharmacies and expanding access via telehealth in US states without abortion bans.^10^ Currently, the consent form requires only the woman’s signature.

France and China were the first countries to authorize mifepristone. Given the FDA’s reputation, US approval accelerated global adoption; today, mifepristone is approved in over 90 countries.^11^ Its use has also grown in the US, where, in 2023, medical abortions accounted for over 60% of all abortions before 10 weeks of gestation.^12^

## CONCLUSION

There is no evidence that prior medical abortion, compared with surgical abortion, increases the subsequent risk of spontaneous abortion, ectopic pregnancy, preterm birth, or low birth weight.^13^ In addition, medical abortion has not been associated with increased risks of breast cancer, mental health disorders, infertility, or subsequent pregnancy loss.^8^

The development of antiprogestin therapy marked a breakthrough in female reproductive endocrinology. It has been hailed as one of the 20th century’s most significant advancements in women’s health.^14^

It has now been over four decades since mifepristone, the first progesterone antagonist, was introduced.^15^ Unfortunately, its association as an abortifacient appears to have limited its exploration in other therapeutic contexts. The potential clinical benefits of mifepristone outside pregnancy remain underexplored, although other antiprogestins—those not used as abortifacients—are now being tested in non-pregnancy-related conditions.

## Abbreviations

FDA: Food and Drug Administration (US)
RU-486: mifepristone
US: United States
